# Spin caloritronics of a quantum dot coupled to a magnetic insulator and normal metal

**DOI:** 10.1038/s41598-025-04413-6

**Published:** 2025-07-02

**Authors:** Emil Siuda, Piotr Trocha

**Affiliations:** Institute of Spintronics and Quantum Information, Faculty of Physics and Astronomy, Adam Mickiewicz University, Poznań, 61-614 Poland

**Keywords:** Spintronics, Nanoscale devices, Electronic and spintronic devices

## Abstract

We investigate the generation of spin current through temperature gradients in a quantum dot-based hybrid system. In particular, we study a quantum dot coupled to a magnetic insulator and a (non)magnetic metallic electrode. Generally, each electrode is maintained at a different temperature, resulting in a finite spin current. In this system, spin current of the magnonic type is converted into electric spin current, and *vice versa*, depending on the direction of the temperature gradient. We examine the influence of the magnonic energy-dependent density of states and many-body magnon interactions on thermally induced spin current. Additionally, the system can work as a spin Seebeck engine, converting heat into spin current. We establish the conditions under which the system operates as a heat engine and present results on its performance. Furthermore, we propose a device based on the system that acts as an efficient thermal spin-diode–allowing spin current to pass in one direction of the temperature bias while completely suppressing it in the opposite direction.

## Introduction

Since the discovery of the spin Seebeck effect^1^ and the spin Peltier effect^2^, the interconnection between heat, charge, and spin currents at the nanoscale has been the subject of intense study^3–8^. The conversion of heat into spin voltage allows for the renewable utilization of waste heat, which is normally emitted into the environment. In this context, nanoscale objects such as molecular junctions, point contacts, or quantum dots (QDs) coupled to external leads offer advantages over bulk materials, including scalability, tunability (e.g. via gate voltage), and the ability to exploit quantum size and interference effects^9–14^. Moreover, when the leads are ferromagnetic or in the presence of an external magnetic field, thermoelectric transport becomes spin-dependent, making these systems useful for potential applications in spintronics^15–18^.

Magnonics is a subfield of spintronics in which magnons–quanta of spin waves–serve as the carriers of spin current^19^. Spin waves exhibit many beneficial transport properties: they carry angular momentum and energy without the Joule heating typical of spin currents, which are due to spin-polarized electron transport^20^. Furthermore, magnons can propagate over distances of up to several centimeters without scattering, which reduces undesirable heating^21–23^. These properties open the door to efficient and energy-saving computational technologies based on magnons, with many devices proposed to use magnons as signal carriers^24–28^. Spin waves can be generated by various methods, including ultra-short laser pulses^25,29^, induction from microstrip antennas into the propagation medium^30^, or spin-orbit torque at interfaces exploiting the spin Hall effect^31–33^, orbital Hall effect^34–37^, and the Rashba-Edelstein effect^38^.

Another promising method for spin current generation relies on applying a temperature gradient, which leads to a variety of spin thermoelectric effects^39–43^. It has also been shown that thermally driven spin currents in magnetic tunnel junctions exhibit rectification and the negative differential Seebeck effect^44,45^. The amplification of microwaves by heat-to-spin current conversion in magnetic tunnel junctions has also been observed^46^. Additionally, at sufficiently high temperatures, magnon interactions become relevant, leading to surprising phenomena such as the stabilization of a Bose-Einstein condensate^47^ and a change in the sign of the magnon polaron spin Seebeck coefficient^48^. It is important to note that while magnonics offers a promising way to transport information without dissipating heat, electron-based logic still dominates applications. Therefore, there is a need for converters between magnonic and electronic spin currents, which is the primary objective of magnon spintronics^19,20^. Furthermore, since spin current or signal can only be measured indirectly, conversion of spin current into an electric signal is also required, e.g. through the inverse spin Hall effect^49–51^.

QDs, due to their tunable and discrete electronic structure, have proven invaluable in solid-state physics research. In the context of the thermoelectric effects, QDs have shown superb efficiency in heat-to-work conversion^9,14,52^. Furthermore, when a QD is coupled to ferromagnetic leads, the thermoelectric response is modified compared to non-magnetic counterparts^53–55^. Another class of systems is referred to as *hybrid systems*. In hybrid systems, QDs are coupled to leads made of materials that differ in physical properties. This introduces an interplay between different statistics and transport properties of the carriers, resulting in a rich spectrum of transport phenomena. It has been shown that multiterminal systems containing metallic leads and additional ferromagnetic insulators, superconductors, or topological insulators can serve as efficient heat engines^56–64^. The thermoelectric properties of QDs connected to two normal metals and one end of a nanowire have been proposed as a probe for Majorana bound states^65,66^. Hybrid systems can also serve as converters between magnonic spin currents and electronic spin currents^67,68^.

In this work, we investigate the influence of the energy dependence of the magnonic density of states and magnon-magnon interactions on transport through a hybrid system consisting of a QD coupled to a magnetic insulator (a reservoir of magnons) and a ferromagnetic metal (a reservoir of electrons). Including these factors introduces an energy-dependent coupling of the dot to the magnonic reservoir, as well as a temperature-dependent magnonic dispersion relation, which modifies the transport properties and improves the model’s accuracy compared to models with a constant density of states^67^. We show that the system can function as a spin current diode under appropriate conditions. Additionally, we calculate the spin power and efficiency of a device operating as a spin Seebeck engine.

The paper is organized as follows: In Sec. Theoretical framework, we present the model of the system and the method used to perform the transport calculations. In Sec. Results and discussion, we provide analysis of the results divided into four parts. In the first part, we introduce the energy-dependent magnonic density of states and discuss the influence of magnon-magnon interactions. In the second part, we present results on thermally generated spin current. In the third part, we predict and explain the spin-diode effect. In the last part, we consider the system operatings as a heat engine, converting heat into spin current. Finally, we conclude our findings in Sec. Conclusion.

## Theoretical framework

**Fig. 1:**
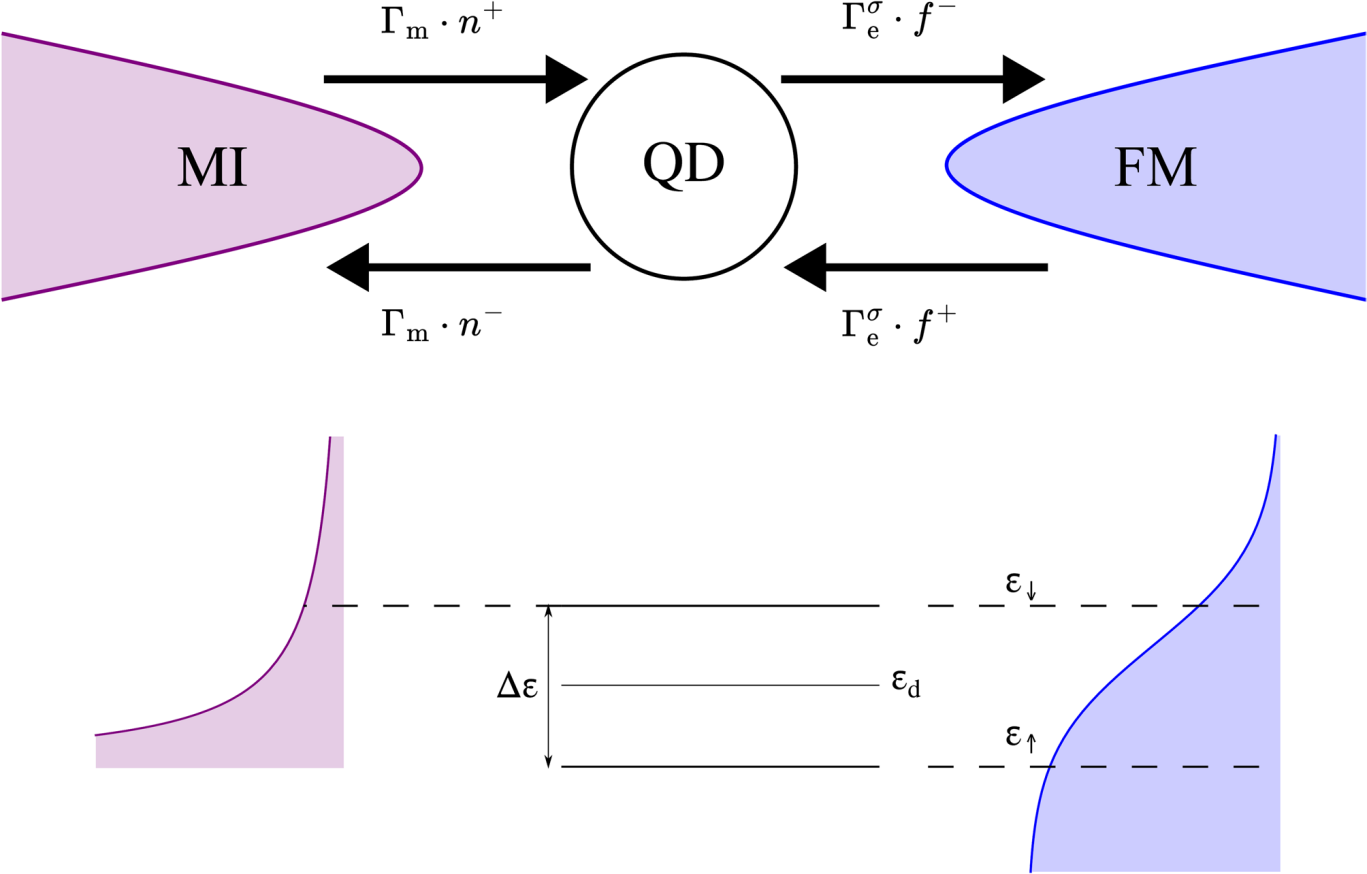
Schematic picture of the system shown in the top panel. It consists of a single-level quantum dot (QD) coupled to a magnetic insulator (MI) and ferromagnetic metal (FM). The level of the QD is split due to the interaction with an external magnetic field $\vec {B}$ directed in the positive *z* direction as shown in the bottom panel. Magnons with the energy equal to $\Delta \varepsilon = g\mu _{\textrm{B}}B$ can be emitted and absorbed by the QD. Simultaneously spin-up and spin-down electrons can tunnel between the dot and the metal. A temperature gradient is present in the system leading to the flow of spin current from the hotter reservoir to the colder one. The tunneling rates are influenced by two factors: coupling strength $\Gamma _{\textrm{m}}$, $\Gamma _{\textrm{e}}^{\sigma }$ between the QD and the leads and number of particles and empty states available in the external leads described by the corresponding distribution functions $n^+$, $f^+$, $n^-$, $f^-$. Here, $\Gamma _{\textrm{m}}n^{+}$ ($\Gamma _{\textrm{m}}n^{-}$) denotes the magnon tunneling rate out of (into) the MI to (from) the QD, while $\Gamma _{\textrm{e}}^{\sigma }n^{+}$ ($\Gamma _{\textrm{e}}^{\sigma }n^{-}$) represents the spin-$\sigma$ electron tunneling rate out of (into) the FM from (to) the QD. In the bottom panel: a Bose-Einstein function (purple) on the left hand side represents the MI, while the Fermi-Dirac distribution of the FM is depicted on the right hand side (blue); black lines denote spin-split QD energy level. Black, dashed lines mark the energies of the corresponding particles contributing to the transport.

### Model

The considered system, presented schematically in Fig. 1, consists of a single-level QD attached to a magnetic insulator (MI) and ferromagnetic metal (FM). The degeneracy of QD’s energy is lifted by the external magnetic field $\textbf{B}$ pointed in the negative *z* direction. The dot can be in one of the four states, denoted as ${|{0}\rangle }$, ${|{\uparrow }\rangle }$, ${|{\downarrow }\rangle }$, ${|{\uparrow \downarrow }\rangle }$, corresponding to the dot being empty, occupied by the electron with spin anti-parallel or parallel to the field, and occupied by two electrons, respectively. The operational principle of the system working as spin current converter can be understand as follows. Let’s assume that the dot’s energy level is situated at the Fermi level of the metallic electrode. An external magnetic field lifts its degeneracy and as a result, it becomes split, i. e. energy of single-occupied dot’s state of an spin-up electron is shifted below the Fermi level by $g\mu _{\textrm{B}}B/2$, while the energy of corresponding spin-down electron is pushed by $g\mu _{\textrm{B}}B/2$ above the Fermi level. By assuming large on-dot Coulomb interactions, the dot can be occupied at least by one electron. Hence, QD coupled to FM electrode is occupied by the electron with spin-up orientation. Moreover, exchange of magnons becomes possible when QD is attached to the ferroMI. Then, the energy of the incoming magnon can be absorbed by a spin-up electron and excite it into the spin-down state. Then, the spin-down electron can tunnel out to the metal, while another spin-up electron tunnels into the free spin-up level of the QD closing the cycle. When the temperature of the magnonic reservoir is higher than that of the metallic electrode, such a cycle is preferable. However, the same process occurs in the opposite direction, but since the tunneling electrons with opposite spins differ in energy, their occupations in the electronic reservoir also vary, which results in the net flow of spin-down electrons into the metal and spin-up electrons out of it. Many such processes lead to an electric spin current flowing out of the metallic electrode. Conversely, reversing the sign of the temperature bias changes the direction of the spin current.

The Hamiltonian describing the system comprises five terms:

1 $$\begin{aligned} H = H_{el} + H_{mag}+H_{QD}+H_{el}^{\textrm{t}}+H_{mag}^{\textrm{t}}. \end{aligned}$$

The first term represents spin-polarized electrons in the metallic lead:

2 $$\begin{aligned} H_{el}=\sum _{{\textbf {k}} \sigma }\varepsilon _{{\textbf {k}} \sigma } c^{\dag }_{ {\textbf {k}} \sigma }c_{ {\textbf {k}} \sigma }, \end{aligned}$$

where $\varepsilon _{{\textbf {k}} \sigma }$ is the energy of electrons with wave vector $\textbf{k}$ and spin $\sigma = \left\{ \uparrow , \downarrow \right\}$ in the metallic lead.

The second term describes the insulating lead, modeled by the Heisenberg Hamiltonian:

3 $$\begin{aligned} H_{mag} = -J_{\textrm{ex}}\sum _{\langle i,j \rangle } \textbf{S}_i \cdot \textbf{S}_j - g_\textrm{m}\mu _\textrm{B}B\sum _i S_i^z, \end{aligned}$$

where $\langle i, j \rangle$ denotes summation over nearest neighbors, $J_{\textrm{ex}}$ ($J_{\textrm{ex}} > 0$) is the exchange integral, and $g_\textrm{m}$ is the Landé factor of the MI. By introducing the raising and lowering operators, $S_i^{\pm } = S_i^x \pm i S_i^y$, and applying the Holstein-Primakoff transformation^69^, the spin operators become:

4 $$\begin{aligned} \begin{aligned} S_i^+&= \sqrt{2S-a_i^{\dag }a_i}a_i\\ S_i^-&= a_i^{\dag }\sqrt{2S-a_i^{\dag }a_i}\\ S_i^z&= S-a_i^{\dag }a_i. \end{aligned} \end{aligned}$$

Expanding the square roots and assuming that $\langle a_i^\dagger a_i \rangle / (2S) \ll 1$, the Hamiltonian $H_{mag}$ can be rewritten using only the lowest-order terms. To account for the contribution of four-magnon interactions to the exchange energy, second-order terms in the expansion of the square roots are retained. For a cubic lattice, the resulting Hamiltonian in Fourier space, within the mean-field approximation, becomes diagonal and reads:

5 $$\begin{aligned} H_{mag} = \sum _{{\textbf {q}}} \varepsilon _{{\textbf {q}}}(T)a^{\dag }_{{\textbf {q}}}a_{{\textbf {q}}}. \end{aligned}$$

Here, $\varepsilon _{{\textbf {q}}}(T)$ is the renormalized energy of magnons^70^, i.e.

6 $$\begin{aligned} \varepsilon _{{\textbf {q}}}(T) = \alpha (T)\bar{\varepsilon }_{{\textbf {q}}}, \end{aligned}$$

with $\bar{\varepsilon }_{{\textbf {q}}}$ being the energy of non-interacting magnons. The temperature-dependent renormalizing factor $\alpha (T)$ is determined by the following equation:

7 $$\begin{aligned} \alpha (T)=1-\frac{1}{2 z J N S^2}\sum _{\textbf{q}'}\frac{1}{\textrm{exp}(\alpha (T)\bar{\varepsilon }_{\textbf{q}'}/[k_{\textrm{B}} T])-1}, \end{aligned}$$

where *N* is the number of particles in the lattice, *z* is the coordination number, *S* is the spin, and $k_{\textrm{B}} T$ is the thermal energy. The magnon dispersion relation is obtained by solving the equation for $\alpha (T)$ self-consistently and substituting the result into $\varepsilon _{\textbf{q}}(T)$. The third term describes the QD:

8 $$\begin{aligned} H_{QD} = \sum _{\sigma }\varepsilon _{\sigma }d^{\dag }_{\sigma }d_{\sigma }+U n_{\uparrow }n_{\downarrow }, \end{aligned}$$

where $\varepsilon _{\sigma } = \varepsilon _{\textrm{d}} \mp \frac{1}{2}g\mu _{\textrm{B}}B$ denotes the Zeeman-split energy level of the dot. Here, minus (plus) sign corresponds to the spin up (down) electron occupying the dot and *U* is the Hubbard parameter indicating the strength of Coulomb repulsion between two electrons residing the dot.

The last two terms describe tunnelling between the QD and the leads. The tunnelling of electrons between the dot and the metallic electrode is described by

9 $$\begin{aligned} H_{el}^{\textrm{t}} = \sum _{{\textbf {k}} \sigma }V_{{\textbf {k}} \sigma }c^{\dag }_{{\textbf {k}} \sigma }d_\sigma + \mathrm {H.c.} \end{aligned}$$

with $V_{{\textbf {k}} \sigma }$ being relevant tunneling amplitude matrix element, while coupling of the dot to MI is given by,

10 $$\begin{aligned} H_{mag}^{\textrm{t}} = \sum _{{\textbf {q}}}j_{{\textbf {q}}}a^{\dag }_{{\textbf {q}}}d^{\dag }_{\uparrow }d_{\downarrow } + \mathrm {H.c.}, \end{aligned}$$

where $j_{\textbf{q}}$ depends on the distribution of interfacial spins and the coupling of the QD to these spins. The Hamiltonian $H_{mag}^{\textrm{t}}$ is derived, similarly as Eq. (5), using the Heisenberg model and the Holstein-Primakoff transformation.

### Method

We investigate spin transport properties of the considered system within a weak coupling regime, i.e. we assume weak coupling between the dot and the reservoirs ($\Gamma _{\textrm{e}}^{\sigma },\Gamma _{\textrm{m}}\ll k_{\textrm{B}}T$) and the Markovian description of transitions between the states of the system. These assumptions allow to employ Pauli master equation method. Let $\textbf{P}$ be a vector of probabilities $P_i$ ($i=0,\uparrow ,\downarrow ,\uparrow \downarrow$) of QD being in a given state i. e. $\textbf{P}=(P_0,P_{\uparrow },P_{\downarrow },P_{\uparrow \downarrow })^T$ and $\textbf{W}$ be a matrix of transition rates between states *i* and *j*. The relevant master equation, governing the vector of probabilities in time, reads:

11 $$\begin{aligned} \frac{d \textbf{P}}{dt} = \textbf{W} \textbf{P}. \end{aligned}$$

In the steady state, Eq. (11) takes form:

12 $$\begin{aligned} \textbf{W} \textbf{P} = 0. \end{aligned}$$

The solution of Eq. (12) together with the normalization of the probabilities, $\sum _i P_i = 1$, provides a unique solutions for the vector $\textbf{P}$. The above equation can be rewritten in the form, $0 = \sum \limits _j W_{ji}P_j - W_{ij}P_i$, with transition rates $W_{ij}$ being given by Fermi golden rule:

13 $$\begin{aligned} W_{ij} = \frac{2\pi }{\hbar }\sum _{mn} \left| \langle n, j\left| H\right| m,i\rangle \right| ^2 w_{\textrm{m}} \delta (E_{m,i}-E_{n,j}) \end{aligned}$$

where ${|{m,i}\rangle }$ is a state of the whole system consisting of the dot in a state ${|{i}\rangle }$ and the leads in a state ${|{m}\rangle }$. The corresponding energy of the state is indicated as $E_{m,i}$. $w_{\textrm{m}}$ is the probability of finding the leads in the initial state ${|{m}\rangle }$. The explicit form of the transition rates matrix is given by:

14 $$\begin{aligned} \textbf{W} = \begin{bmatrix} -\sum _\sigma \Gamma _{\textrm{e}}^{\sigma } f^+_\sigma & \Gamma _{\textrm{e}}^{\uparrow } f^-_\uparrow & \Gamma _{\textrm{e}}^{\downarrow } f^-_\downarrow & 0\\ \Gamma _{\textrm{e}}^{\uparrow } f^+_\uparrow & -\left( \Gamma _{\textrm{e}}^{\uparrow }f^-_\uparrow + \Gamma _{\textrm{e}}^{\downarrow }f^{U,+}_\downarrow + \Gamma _{\textrm{m}}n^+\right) & \Gamma _{\textrm{m}} n^-& \Gamma _{\textrm{e}}^{\downarrow } f^{U,-}_\downarrow \\ \Gamma _{\textrm{e}}^{\downarrow }f^+_\downarrow & \Gamma _\textrm{m} n^+& -\left( \Gamma _{\textrm{e}}^{\downarrow }f^-_\downarrow + \Gamma _{\textrm{e}}^{\uparrow }f^{U,+}_\uparrow + \Gamma _{\textrm{m}}n^-\right) & \Gamma _{\textrm{e}}^{\uparrow } f^{U,-}_\uparrow \\ 0& \Gamma _{\textrm{e}}^{\downarrow } f^{U,+}_\downarrow & \Gamma _{\textrm{e}}^{\uparrow } f^{U,+}_\uparrow & -\sum _\sigma \Gamma _{\textrm{e}}^{\sigma } f^{U,-}_\sigma \end{bmatrix}, \end{aligned}$$

Each off-diagonal element $W_{ij}$ of $\textbf{W}$ is a product of a dot’s coupling strength to the FM electrode, $\Gamma _{\textrm{e}}^{\sigma }$, or to the magnonic reservoir, $\Gamma _{\textrm{m}}$, and the probability that a state with energy matching transition energy is available. $\Gamma _{\textrm{e}}^{\sigma }$ taken in the wide-band approximation is independent of energy and constant. It can be parametrized as follows:

15 $$\begin{aligned} \Gamma _{\textrm{e}}^{\sigma } = \frac{2\pi }{\hbar }\langle \left| V_{\textbf{k}\sigma } \right| ^2\rangle \rho _{\textrm{e}}^{\sigma }=(1 \pm p)\Gamma _\textrm{e}, \end{aligned}$$

where $\rho _{\textrm{e}}^{\sigma }$ is the density of states (DOS) for electrons with spin $\sigma$ in the FM and *p*, defined as $p = \left( \rho _{\textrm{e}}^{\uparrow } - \rho _{\textrm{e}}^{\downarrow } \right) /\left( \rho _{\textrm{e}}^{\uparrow } + \rho _{\textrm{e}}^{\downarrow }\right)$, denotes the corresponding spin polarization factor, and $\Gamma _e=(\Gamma _{\textrm{e}}^{\uparrow }+\Gamma _{\textrm{e}}^{\downarrow })/2$. In contrast, the dot’s coupling to the MI, $\Gamma _{\textrm{m}}$, depends on energy and temperature through the density of states of the MI and takes the form:

16 $$\begin{aligned} \Gamma _{\textrm{m}}(\varepsilon _{{q}},T_{\textrm{m}})=\frac{2\pi }{\hbar }\langle \left| j_{\textbf{q}} \right| ^2\rangle \rho _{\textrm{m}}(\varepsilon _{{q}},T_{\textrm{m}})\equiv j_{0}\rho _{\textrm{m}}(\varepsilon _{q},T_{\textrm{m}}), \end{aligned}$$

where the energy and temperature dependencies are explicitly indicated. The temperature dependence arises from magnon-magnon interactions; in models that neglect these interactions, only energy dependence remains. To simplify the notation, we write $\Gamma _{\textrm{m}}(\epsilon _{\vec {q}}, T_{\textrm{m}}) \equiv \Gamma _{\textrm{m}}$, and whether magnon-magnon interactions, i.e. the temperature dependence of $\Gamma _{\textrm{m}}$, are included or omitted will be clearly stated. Note that previous works have not considered such an energy dependence, assuming a wide-band approximation also with respect to the QD coupling with the MI^67,71^. In Eqs. (15) and (16) $\langle \left| V_{\textbf{k}\sigma } \right| ^2\rangle$ and $\langle \left| j_{\textbf{q}} \right| ^2\rangle \equiv j_0$ denote averages over wave vectors $\textbf{k}$ and $\textbf{q}$, respectively. The magnonic density of states $\rho _{\textrm{m}}(\varepsilon _{\vec {q}},T_{\textrm{m}})$ can be obtained directly from the relevant dispersion relation as is explicitly derived in Sec. MI density of states.

The probabilities of finding particles and free states needed for the corresponding transition to happen are given by the appropriate distribution function: the Fermi - Dirac distribution $f_{\sigma }^{[U,]\pm }(\varepsilon = \varepsilon_\sigma \ [+ U])\equiv f_{\sigma }^{\pm }$ for electrons and Bose-Einstein distribution $n^{\pm }(\varepsilon = g\mu_BB)\equiv n^{\pm }$ for magnons. The distribution functions are given as:

17 $$\begin{aligned} f_{\sigma }^+(\varepsilon )&= \left[ \textrm{exp}\left( \frac{\varepsilon -\mu _{\sigma }}{k_\textrm{B} T_\textrm{e}}\right) +1\right] ^{-1}\equiv 1 - f_{\sigma }^-(\varepsilon ),\end{aligned}$$

18 $$\begin{aligned} n^+\left( \varepsilon \right)&= \left[ \textrm{exp}\left( \frac{\varepsilon }{k_\textrm{B} T_\textrm{m}}\right) -1\right] ^{-1}\equiv n^-(\varepsilon )-1, \end{aligned}$$

where $T_{\textrm{e}(\textrm{m})}$ stands for the temperature of an electronic (magnonic) reservoir and $\mu _{\sigma }$ denotes the chemical potential in the metallic electrode for electrons with spin $\sigma$. Here, we define temperatures of left and right electrodes in the following way:

19 $$\begin{aligned} \begin{aligned}&T_{\textrm{m}} = T_0 + \Delta T/2\\&T_{\textrm{e}} = T_0 - \Delta T/2, \end{aligned} \end{aligned}$$

where $T_0$ is the mean temperature and $\Delta T$ is a temperature difference set between the reservoirs, $\Delta T=T_\textrm{m}-T_\textrm{e}$. Notice, that chemical potential in the FM electrode is assumed to be spin-dependent, which allows to form the spin bias voltage, $V_s$. Specifically, we incorporate spin bias voltage into spin-dependent chemical potential as follows,

20 $$\begin{aligned} \mu _{\sigma }=\mu +\tilde{\sigma }\frac{\Delta \mu _\textrm{s}}{2} \end{aligned}$$

with $\tilde{\sigma }=1$ ($\tilde{\sigma }=-1$) for $\sigma =\uparrow$ ($\sigma =\downarrow$), $\mu$ denoting chemical potential and $\Delta \mu _\textrm{s}\equiv eV_\textrm{s}=\mu _{\uparrow }-\mu _{\downarrow }$. Spin bias leads to spin-dependent Fermi-Dirac function, which was explicitly indicated in Eq. (17).

### Spin and heat current

The total spin current $J_\textrm{s}$ flowing to the QD includes both magnonic and electronic contributions, $J_{\textrm{m}}$ and $J_\textrm{e}$, respectively, and thus $J_\textrm{s} = J_{\textrm{e}}+J_{\textrm{m}}$. In the steady-state, spin angular momentum conservation gives

21 $$\begin{aligned} J_{\textrm{e}}+J_{\textrm{m}}= \frac{\hbar }{2}\left( \dot{P}_{\uparrow }-\dot{P}_{\downarrow }\right) =0, \end{aligned}$$

and hence, $J_{\textrm{m}}=-J_\textrm{e}$. The spin current flowing from magnonic reservoir can be calculated using the formula

22 $$\begin{aligned} J_{\textrm{m}} = -\hbar (P_{\uparrow }W_{\uparrow \downarrow }-P_{\downarrow }W_{\downarrow \uparrow }) = -\hbar I_{\textrm{m}}, \end{aligned}$$

where $I_{\textrm{m}}$ stands for the magnon current For positive temperature bias ($\Delta T > 0$), the magnon current is positive ($I_{\textrm{m}}>0$) as magnons flow from magnonic reservoir to the dot. In contrast, negative magnon current is expected for $\Delta T < 0$.

The heat current flowing through the system is a flux of energy carried along with the magnon current

23 $$\begin{aligned} J_{Q} = \varepsilon _{\textrm{q}}I_{\textrm{m}}, \end{aligned}$$

where $\varepsilon _{\textrm{q}} = g \mu _{\textrm{B}} B$ is the energy of the magnon transferred through the QD. Here we consider the situation with $B>0$, hence positive heat current is associated with the magnon current flowing out of the magnonic reservoir.

The system can work as spin-heat engine, converting the heat into spin current. Hence, we introduce the quantities that describe the performance of the engine. Namely, the output power of the spin current defined as work done by spin current per unit time, is given by

24 $$\begin{aligned} P = -I_{\textrm{m}} \Delta \mu _s. \end{aligned}$$

is equal to heat currents exchanged between the QD and the reservoirs. The corresponding efficiency is expressed by

25 $$\begin{aligned} \eta = \frac{P}{J_Q} \end{aligned}$$

where $J_Q$ is positively defined only if $T_{\textrm{m}}>T_{\textrm{e}}$.

## Results and discussion

This section is structured into several parts. First, we present the dispersion relation and the density of states (DOS) of the MI, followed by an analysis of how magnon interactions influence these quantities. Next, we discuss the results related to the magnon (spin) current, emphasizing the role of magnon interactions. These findings are compared with those obtained in the long-wavelength limit, and the impact of the energy-dependent DOS of the MI is examined. Additionally, we explore the effects of the magnetic field and the spin polarization of the ferromagnetic (FM) electrode. Subsequently, we introduce the conditions under which the system works as *spin diode*. In the last section, we examine the device operating as a spin heat engine and derive the condition under which it works with the best efficiency.

In the numerical calculations we assume the magnonic reservoir is made of yttrium-iron-garnet (YIG) with the following parameters taken from the literature ^72^: lattice constant $a = 1.2378$ nm ^73^, frequency of acoustic magnons at the edge of the Brillouin zone $E_{\textrm{BZ}}/h = 8$ THz, magnitude of the wave vector at the edge of the Brillouin zone $q_{\textrm{BZ}} = 2.8/a$, exchange integral $J_{\textrm{ex}} = 0.82$ meV, Lande factor $g_{\textrm{m}} = 2$ ^74^, coordination number $z = 6$, atomic spin $S = 5/2$. Furthermore, we assume $j_0 = 10^{-33} \ \mathrm {eV \ m^3/s}$ ensuring weak coupling between the dot and the MI. The dot’s coupling to the FM electrode is assumed to fulfill the relation $\Gamma _{\textrm{e}}^{\sigma }\ll k_{\textrm{B}}T$. The dot’s energy level is set at $\varepsilon _d=0$, and infinite Coulomb interactions are assumed $U\rightarrow \infty$ unless stated otherwise.

### MI density of states and the role of magnon-magnon interactions

To perform analytical calculation of the MI density of states, we make an approximation of the dispersion relation introduced in Sec. Model, given by Eq. (6). In particular, we approximate the cubic Brillouin zone of YIG by a sphere containing most of the momentum states

26 $$\begin{aligned} \bar{\varepsilon }_q = E_{\textrm{BZ}}\left[ 1-\cos \left( \frac{\pi }{2}\frac{q}{q_{\textrm{BZ}}}\right) \right] \end{aligned}$$

and refer to it as the *spherical (*SP*) model*. Here, *q* is the magnitude of the wavevector $\textbf{q}$. For this energy spectrum, we calculate the DOS of the magnonic reservoir. Since there is no known analytical solution to Eq.  (7), numerical methods or simplifications must be employed to account for the influence of the magnon-magnon interactions for the DOS.

**Fig. 2:**
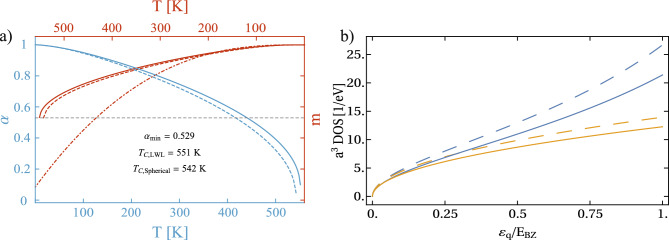
(**a**) Energy renormalization factor $\alpha$ (blue lines) calculated self-consistently within LWL model (solid line), SP model (dashed line), with Eq. (28) (dot-dashed line) and reduced magnetization *m* (red lines) calculated self-consistently within LWL model (solid line) and SP model (dashed line) as a function of the temperature. (**b**) DOS as a function of magnon’s energy. Solid lines indicate no magnon - magnon interaction ($\alpha = 1$) and dashed lines are for $\alpha$ calculated self-consistently within LWL (orange) and SP (blue) models for the temperature $T = 250$ K.

Before presenting the DOS, we introduce the long wavelength limit (LWL) of the dispersion relation given by SP [Eq. (26)]. In the LWL, only the lowest energy states with large wavelengths (small frequencies) are considered. Under this assumption, one can expand Eq. (26) into a series and keep the terms up to the quadratic order in *q*, which results in

27 $$\begin{aligned} \bar{\varepsilon }_{q} = \frac{E_{\textrm{BZ}} \pi ^2}{8}\left( \frac{q}{q_{\textrm{BZ}}} \right) ^2. \end{aligned}$$

The magnon-magnon interactions are accounted for by the factor $\alpha (T)\equiv \alpha$, introduced by Eq. (7), which has to be solved in a self-consistent way, as no analytical solution exists. One can calculate $\alpha$ numerically within SP or LWL model by solving Eq. (7). However, assuming LWL model, a crude approximation can be done, by putting $\alpha =1$ on the right hand side of Eq. (7). This leads to the following approximate analytical formula^44^:

28 $$\begin{aligned} \alpha = 1 - \frac{3a^3q_{\textrm{BZ}}^3\zeta \left( \frac{5}{2}\right) }{\sqrt{2}zJ_{\textrm{ex}}S^2 E_{\textrm{BZ}}^{3/2}}\left( k_{\textrm{B}}T\right) ^\frac{5}{2}, \end{aligned}$$

where $\zeta \left( \cdot \right)$ is Riemann zeta function. We also calculated reduced magnetization for the introduced dispersion models using formula,

29 $$\begin{aligned} m\left( T\right) = \frac{M\left( T\right) }{M\left( 0\right) } = 1 - \frac{1}{NS}\sum _{q}\left<a^{\dagger }_{q}a_{\textbf{q}}\right>. \end{aligned}$$

Having established the models for the dispersion relation and clarified the calculations regarding the interaction factor $\alpha$ we are ready to derive the corresponding DOS. The density of states is calculated using standard definition,

30 $$\begin{aligned} \rho (\varepsilon ) = \frac{2\Omega _{d-1}}{(2\pi ^d)}\int _{0}^{\infty }\delta \left( \varepsilon - \varepsilon _{q} \right) q^d \textrm{d}q, \end{aligned}$$

where *d* is the number of dimensions of the sample, and $\Omega$ denote a solid angle of the *d*-dimensional sphere. Using equations (26) and (27), we obtain DOS

31 $$\begin{aligned} \rho _{\textrm{SP}} = \frac{4 q_{\textrm{BZ}}^3 \left[ \cos ^{-1}\left( 1-\frac{\varepsilon _{\textrm{q}}}{E_{\textrm{BZ}}}\right) \right] ^2}{\pi ^5\sqrt{2\varepsilon _{\textrm{q}}E_{\textrm{BZ}}-\varepsilon _{\textrm{q}}^2}} \end{aligned}$$

for the spherical model and

32 $$\begin{aligned} \rho _{\textrm{LWL}} = \frac{2^{3/2}q_{\textrm{BZ}}^3}{E_{\textrm{BZ}}^{3/2}\pi ^5}\sqrt{\varepsilon _{\textrm{q}}} \end{aligned}$$

for the LWL model. In the limit of $\varepsilon \rightarrow 0$, DOS described by both formulas vanishes, indicating that zero-energy magnons cannot be excited. This aligns with the expectations and stands in stark contrast to the wide-band approximation^67,71^.

Now let us present numerical calculations regarding the introduced quantities. Figure 2 a) shows the temperature dependence of the energy renormalization factor $\alpha$ and the reduced magnetization *m* calculated for the SP and LWL models. First, magnon interactions become significant at sufficiently high temperatures and vanish completely in the zero-temperature limit, as expected. Second, both SP and LWL models predict quite similar temperature dependence of $\alpha$. Slight differences occur at higher temperatures and become increasingly pronounced as the temperature rises. In contrast, the interaction factor calculated using the approximate analytical formula, Eq. (28), agrees with the other two only at temperatures up to approximately 200 K but diverges increasingly as the temperature rises further, overestimating the magnon-magnon interactions. Both SP and LWL models predict a Curie temperature close to the experimental one, being around $549-560$ K ^72^.

Figure 2 b) presents the influence of magnon-magnon interactions on the DOS calculated with the SP and LWL dispersion relations. The numerical calculations confirm that the DOS vanishes as the energy of the magnons approaches zero. In both cases, magnon interactions lead to an enhancement of the DOS, which becomes increasingly pronounced with increasing magnon energy. Moreover, the difference between the DOS within the SP and LWL models becomes more relevant for larger energies. This difference exists independently of the temperature. In contrast, magnon interactions are relevant only at sufficiently high temperatures, as shown in additional figures provided in the Supplementary Information (SI).

### Spin current of interacting magnons

After performing calculations described in Sec. Spin and heat current, the spin current takes the form:

33 $$\begin{aligned} J_{\textrm{m}} = -\hbar \frac{\Gamma _{\textrm{m}}(\varepsilon )\Gamma _{\textrm{e}}^{\uparrow }\Gamma _{\textrm{e}}^{\downarrow }\left( f_{\downarrow }^-f_{\uparrow }^+n^+-f_{\downarrow }^+f_{\uparrow }^-n^-\right) }{\Gamma _{\textrm{e}}^{\uparrow }\Gamma _{\textrm{e}}^{\downarrow }\left( f_{\downarrow }^+f_{\uparrow }^-+f_{\downarrow }^-f_{\uparrow }^++f_{\downarrow }^-f_{\uparrow }^-\right) +\Gamma _{\textrm{e}}^{\uparrow }\Gamma _{\textrm{m}}(\varepsilon )\left( f_{\uparrow }^-n^-+f_{\uparrow }^+n^-+f_{\uparrow }^+n^+\right) +\Gamma _{\textrm{e}}^{\downarrow }\Gamma _{\textrm{m}}(\varepsilon )\left( f_{\downarrow }^+n^-+f_{\downarrow }^+n^++f_{\downarrow }^-n^+\right) } \end{aligned}$$

in the $U\rightarrow \infty$ limit. Here, we explicitly denote the energy dependence of the dot’s coupling to the magnonic reservoir but omit it in the Fermi and Bose functions for conciseness. Namely, in the above notation, one has $f_{\sigma }^{+/-}\equiv f_{\sigma }^{+/-}(\varepsilon =\varepsilon _{\sigma })$ and $n^{+/-}\equiv n^{+/-}(\varepsilon =g\mu _{\textrm{B}}B)$.

**Fig. 3:**
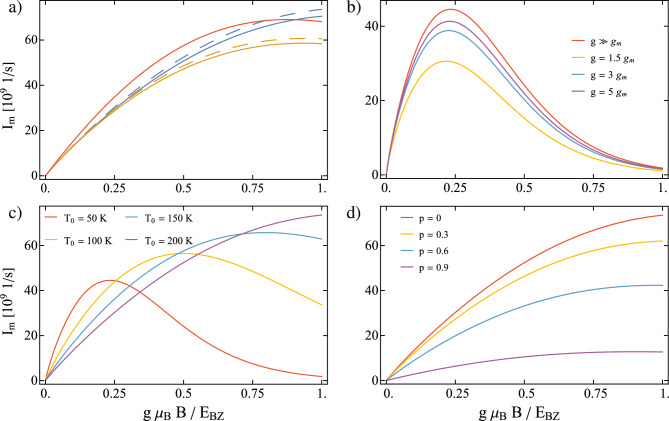
(**a**) Magnon current as a function of $g\mu _{\textrm{B}}B$ for $T_0 = 200$ K, $\Delta T = 0.1 T_0$, $g \gg g_m$ and $p = 0$. Solid lines indicate no magnon - magnon interaction ($\alpha = 1$) and dashed lines are for $\alpha$ calculated self consistently. For comparison, the red curve is current in the broadband approximation when $\rho _{\textrm{m}} = const$ (**b**), for indicated values of the QD’s Lande factor, $p = 0$, $T_0 = 50$ K and $\Delta T =0.1T_0$ c), for indicated values of $T_0$, $\Delta T = 0.1T_0$, $g \gg g_m$ and $p = 0$ d), for indicated values of *p*, $T_0 = 200$ K, $g \gg g_m$ and $\Delta T = 0.1T_0$. The other parameters are as in Fig. 2.

Note that QD filters only magnons with energy equal to $\varepsilon =g\mu _{\textrm{B}}B$, which is clearly indicated in the energy dependence of $\Gamma _{\textrm{m}}(\varepsilon )$ via the relevant density of states. By changing the magnetic field, one tunes the splitting of the dot’s level, and thus, the energy of the transmitted magnons. Figure 3 a) presents the magnon current, $I_{\textrm{m}}=-J_{\textrm{m}}/\hbar$, as a function of energy of the transported magnons. The magnon current was calculated for both the SP and LWL dispersion relations. Additionally, the cases with and without interactions between magnons were considered for both models. In general, magnon-magnon interactions tend to increase the spin current due to the higher DOS, resulting from temperature dependent dispersion relation shown in Fig. S3 b) in the SI. However, the influence of these interactions becomes relevant only for magnons with sufficiently high energies. For comparison, the magnon current calculated for the LWL model and by using approximation Eq. (28) is also shown. In general, this approximation leads to an overestimation of the $I_{\textrm{m}}$, especially at lower temperatures, as shown in Fig. S2 in the SI. However, an increase in temperature reduces this discrepancy, and for sufficiently high temperatures, qualitatively and roughly quantitatively follows the $I_{\textrm{m}}$ obtained within the SP and LWL models. For $I_{\textrm{m}}$ calculated at different temperatures, we refer the reader to the Supplementary Information. In turn, the differences between the magnon current calculated within the LWL and SP models become increasingly pronounced with increasing of magnon energy. After clarifying the differences between the various models, the forthcoming results are obtained only for the SP model with the inclusion of magnon interactions, as this covers full parameter space.

Up to now, we have neglected the influence of the magnetic field on the MI and the corresponding dispersion relation (26) by assuming that its *g*-factor is much smaller than the *g*-factor associated with the QD. Taking into account the contribution from the interaction with the external magnetic field, the dispersion relation takes the form

34 $$\begin{aligned} \bar{\varepsilon }_q = E_{\textrm{BZ}}\left[ 1-\cos \left( \frac{\pi }{2}\frac{q}{q_{\textrm{BZ}}}\right) \right] + g_{\textrm{m}} \mu _{\textrm{B}} B, \end{aligned}$$

where $g_{\textrm{m}}$ is the Landé factor of the MI. This causes a horizontal shift in the DOS, opening a gap as presented in Fig. S2 a) in the SI.

Figure 3 b) compares the magnon current calculated with the dispersion relations (26) and (34) for different values of *g* expressed in units of $g_{\textrm{m}}$. Here, *g* is tuned, whereas $g_{\textrm{m}}=2$ is fixed. The smaller the difference between *g* and $g_{\textrm{m}}$, the smaller the magnon current, eventually vanishing entirely when $g<g_{\textrm{m}}$. The decrease in magnon current with decreasing *g* can be understood by referring to the dispersion relation and the presence of a gap when the magnetic field is applied. Specifically, as *g* decreases, the energies ($g\mu _{\textrm{B}}B$) of transmitted magnons also diminish. At the same time, for a given *B* and *T*, fewer magnons can be excited to states above the energy gap compared to the situation where the Zeeman term in the dispersion relation is neglected. Finally, for $g < g_{\textrm{m}}$, there are no magnons with energies of $g\mu _{\textrm{B}}B$ in the MI, and thus, the magnon current is completely suppressed. The suppresion of the magnon current with decreasing dot’s *g* factor can also be explained by referring to the conservation of energy, which reads

35 $$\begin{aligned} \bar{\varepsilon }_{q} = \Delta \varepsilon \equiv g\mu _{\textrm{B}}B \end{aligned}$$

or, taking the explicit form of the dispersion relation

36 $$\begin{aligned} E_{\textrm{BZ}}\left[ 1-\cos \left( \frac{\pi }{2}\frac{q}{q_{\textrm{BZ}}}\right) \right] + g_{\textrm{m}} \mu _{\textrm{B}} B = g \mu _{\textrm{B}} B. \end{aligned}$$

When *g* approaches $g_{\textrm{m}}$, the first term on the left-hand side of Eq. (36) must approach zero for the equality to hold, which also means that *q* tends to zero. In turn, as *q* aproaches zero, fewer magnons are available to conduct the magnon current. This also explain the assumed condition for the relation between *g* and $g_{\textrm{m}}$, namely $g\ge g_{\textrm{m}}$. On the other hand, neglecting the influence of the magnetic field on the magnons in MI is equivalent to the assumption, $g \gg g_{\textrm{m}}$ which is held in the forthcoming calculations, as the results can change only quantitatively.

In Fig. 3 c), we present the influence of the temperature on the magnon current. The current attains a maximum which shifts toward higher magnon energies as the temperature increases, up to the point when the maximum approaches the cut-off energy for acoustic magnons, i.e. for $g\mu _{\textrm{B}}B > E_{\textrm{BZ}}$, which occurs for sufficiently high temperatures. An increase in the amplitude of $I_{\textrm{m}}$ and shift of its maximum towards higher *B* with $T_0$ occurs because more high-energy magnons are excited in the MI, and more magnons overall are available for transport.

Figure 3 d) presents the influence of the spin polarization of the FM electrode on $I_m$. The increasing shortage of minority carriers bottlenecks the magnon current, leading to its decline with higher values of spin polarization. Note that electrons with both spin orientations must exist in the FM electrode to create spin-flip proccess on the dot. Thus, the magnon current is maximized for a nonpolarized metallic electrode, i.e. for $p=0$, and is totally suppressed for $p=1$.

### Rectification of the current and spin diode effect

**Fig. 4:**
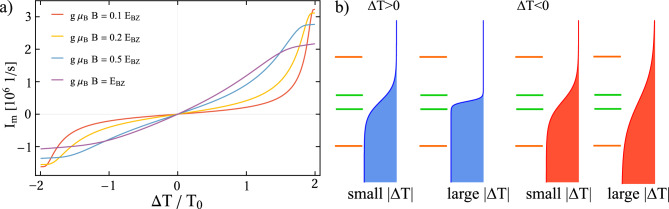
Magnon current as a function of the temperature difference between the reservoirs calculated for indicated values of the magnetic field *B*. The schematic picture in b) supports the explanation presented in the main text. The blue-colored Fermi function on the left represents the FM electrode for a positive temperature bias, $\Delta T > 0$, while the red Fermi distribution on the right corresponds to $\Delta T < 0$. In both cases, there are two profiles of the Fermi function corresponding to small and large values of $|\Delta T|$. The green (orange) horizontal lines represent the dot’s energy level, split by a small (large) magnetic field. The other parameters are as in Fig. 2 and $T_0 = 200$ K.

In this section, we demonstrate that the system can function as a diode, rectifying thermally generated spin current. Figure 4 presents the magnon current as a function of the temperature differences $\Delta T$ applied to the reservoirs. In Fig. 4 a), the absolute value of the magnon current rises with increasing magnetic field *B* for certain range of $\Delta T$, beyond which the opposite behavior is observed as a consequence of competition between Bose-Einstein and Fermi-Dirac distribution of particles. To explain this behavior in more detail, let us first consider a small magnetic field *B* and a positive temperature bias ($\Delta T > 0$). For $0<\Delta T < T_0$, the magnon current increases slowly and roughly linearly with $\Delta T$, which results from the distribution of electrons around the Fermi level of the FM electrode within the range of $g\mu _{\textrm{B}}B$. Although there are plenty of magnons in the MI reservoir with energies $g\mu _{\textrm{B}}B$ high enough to excite the electron on the QD, the number of electrons (empty states) with energies $\varepsilon _{\uparrow } = -g\mu _{\textrm{B}}B/2$ ($\varepsilon _{\downarrow } = g\mu _{\textrm{B}}B/2$) below (above) the Fermi level is relatively small [schematically shown in the first picture in Fig. 4 b)], resulting in a small magnon current. For sufficiently large $\Delta T$ [refer to the second picture in Fig. 4 b) ], the temperature of the FM lead becomes low enough to provide a large number of electrons (empty states) with energies $-g\mu _{\textrm{B}}B/2$ ($g\mu _{\textrm{B}}B/2$), causing the magnon current to grow rapidly with further increases in $\Delta T$. In contrast, for large magnetic fields, the Fermi-Dirac distribution at energies $-g\mu _{\textrm{B}}B/2$ and $g\mu _{\textrm{B}}B/2$ readily allows electron transitions between the dot and the electronic reservoir, facilitating spin-flip processes. As a result, even for small $\Delta T$, a significant magnon current is observed.

On the other hand, the decrease in magnon current with increasing *B* for sufficiently large $\Delta T$ can be explained by the temperature dependence of the Bose-Einstein distribution. Although the temperature of the FM electrode is low enough that the Fermi-Dirac distribution permits nearly the same electron transition rates between the QD and the metallic lead for both small and large *B* [refer to the second picture in Fig. 4 b)], the faster increase in the population of low-energy magnons compared to high-energy magnons with increasing $\Delta T$ leads to the observed magnon current behavior.

Moreover, the magnon current $|I_{\textrm{m}}|$ is not symmetric with respect to the reversal of the temperature bias. This rectification arises from the temperature dependence of the Bose-Einstein distribution. Specifically, an increase in $\Delta T$ raises the temperature of the MI reservoir, increasing the population of magnons with energies high enough to excite electrons on the dot, as described earlier. Conversly, a reversal of $\Delta T$ lowers the temperature of the MI reservoir, resulting in a smaller number of magnons that can be absorbed by the MI. Note that for $\Delta T < 0$, electron transitions between the QD and the FM electrode lead to magnon emission by the dot, which are then absorbed by the magnonic reservoir. Additionally, as $\Delta T$ decreases further, the Fermi-Dirac distribution increasingly facilitates these transitions as inferred from the third and fourth scheme presented in Fig. 4 b). Furthermore, magnon interactions enhance rectification, as the magnon current is significantly increased by these interactions at high MI temperatures.

**Fig. 5:**
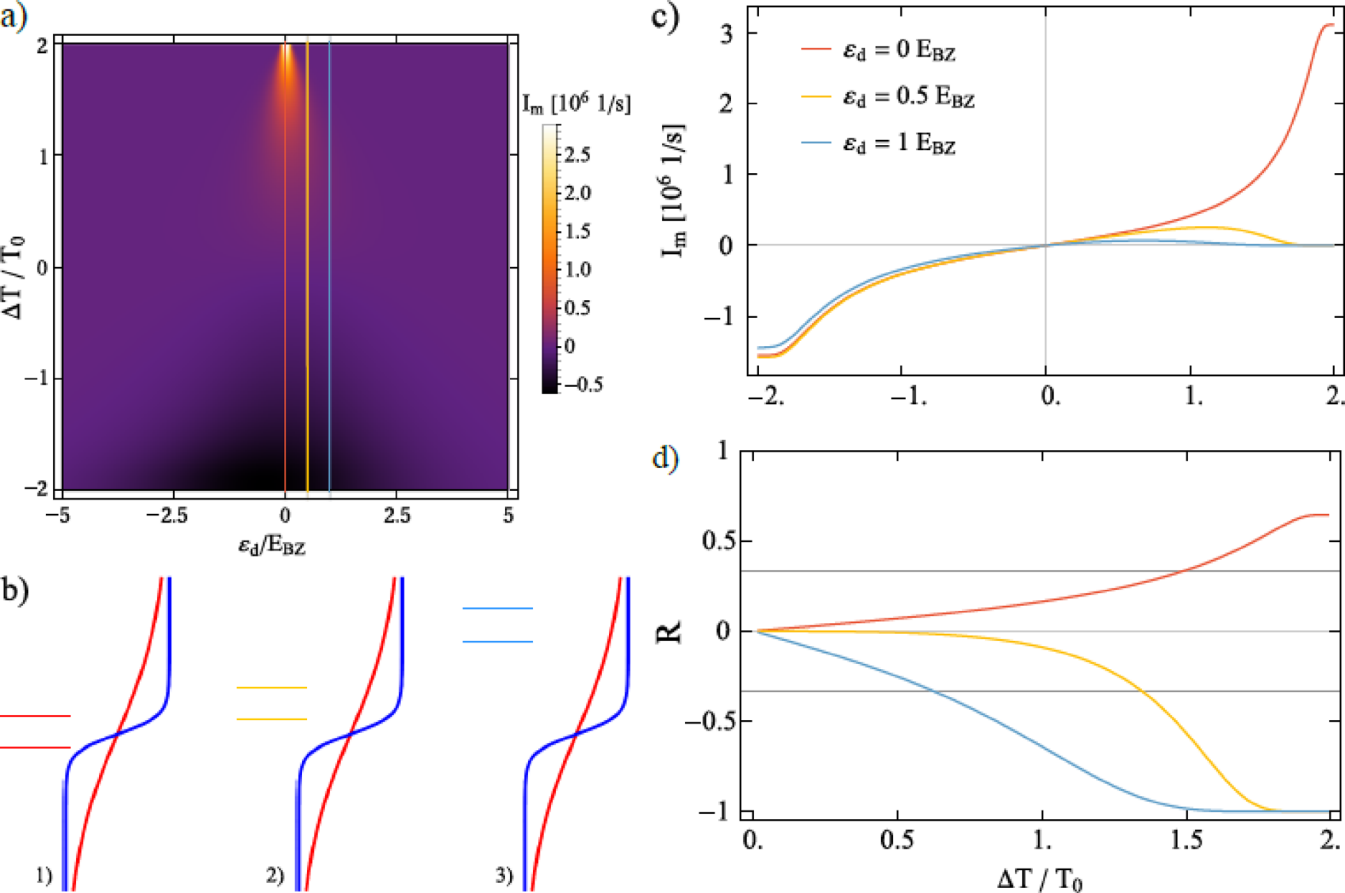
(**a**) Magnon current as a function of the temperature difference and the dot’s level position. Red, yellow and blue lines indicate energy level for which: Fermi profiles, magnon current and rectification are presented with corresponding colours in the panels (**b-d**). (**b**) Fermi profiles colored in blue (red) for positive (negative) temperature bias. The dot’s energy level split by magnetic field is represented by colored horizontal lines; red, yellow, blue lines refers to increasing values of $\varepsilon _d$, which are indicated by the corresponding lines in (**a, c, d**). Panel (**c**) presents magnon current as a function of the temperature difference set to the reservoirs. Panel (**d**) presents rectification as a function of the temperature difference set to the reservoirs. The grey lines represent $R = \frac{1}{3}$, for which $I_{\textrm{m}} (-\Delta T) = \pm 2I_{\textrm{m}}(\Delta T)$. The parameters are $T_0 = 200$ K, $g \mu _{\textrm{B}} B = 0.2 E_{\textrm{BZ}}$. The other parameters are as in Fig. 2.

In Fig. 5 a), we show magnon current as a function of the temperature bias and the dot’s energy level. In Fig. 5 c), the corresponding cross-sections indicated by the color lines in Fig. 5 a) are presented. Fig. 5 c) clearly shows strong rectification of the magnon current when the dot’s energy level is far above the Fermi level of the FM electrode. Specifically, the magnon current is strongly suppressed for $\Delta T>0$ and reaches non-zero values when the temperature bias is reversed. This can be explained by considering the Fermi-Dirac distribution profiles shown in Fig. 5 b) and the argumentation presented earlier. As the dot’s energy level moves away from the Fermi energy of the FM lead, the number of electrons available for transport decreases. For $\Delta T > 0$, when $\varepsilon _d$ is situated far above the Fermi level of the FM electrode, the electron transition rates between the QD and the FM reservoir are suppressed, as only a small population of electrons is available in the metallic lead. Moreover, although a large population of magnons is expected in the MI for large $\Delta T$, these magnons cannot be transferred through the dot due to lack of electrons with spin-up in the FM. In contrast, the magnon current for negative temperature bias decreases only slightly as $\varepsilon _d$ increases moving away from the Fermi level. The corresponding Fermi distribution, which now changes slowly with energy, is depicted in red in Fig. 5 b). Even for relatively large values of $\varepsilon _d$, there are still plenty of electrons in the FM electrode that can tunnel to the dot, allowing magnon emission to MI. As a consequence, the temperature bias dependence of the magnon current results in a spin-diode effect for $\varepsilon _d$ far away from the Fermi level. To support the above results more quantitatively, we calculate the rectification factor, defined as

37 $$\begin{aligned} R = \frac{\left| I_{\textrm{m}}(\Delta T)\right| -\left| I_{\textrm{m}}(-\Delta T)\right| }{\left| I_{\textrm{m}}(\Delta T)\right| +\left| I_{\textrm{m}}(-\Delta T)\right| } \end{aligned}$$

where $I_{\textrm{m}}(\Delta T)$ denotes the magnon current calculated for temperature difference $\Delta T$. This definition constrains the rectification factor to values within the range $\langle -1,1 \rangle$. When $R=0$, there is no rectification, while $R=1$ or $R=-1$ indicates complete rectification of the magnon current. Positive values of *R* mean that the magnon current for a positive $\Delta T$ is greater than for the corresponding negative $\Delta T$, whereas negative *R* signifies the opposite. Figure 5 d) presents rectification factor *R* corresponding to the currents presented in the Fig. 5 c) Furthermore, the diode effect is very robust under varying system parameters as demonstrated in Fig. S4 in the SI.

### Power output and efficiency

The system can also work as a nanoscale engine, converting heat into spin current. The output power can be extracted when a spin voltage $V_s$ ($\Delta \mu _s=eV_s$) is applied, against which the thermoelectric spin current can do work. The system under consideration has been shown to work with Carnot efficiency in the linear response regime^68^. Here, we assume $T_{\textrm{m}} > T_{\textrm{e}}$, which is associated with a positive magnon current. To find the boundary between positive and negative magnon current, one needs to solve the equation $I_{\textrm{m}} = 0$, which results in:

38 $$\begin{aligned} g\mu _{\textrm{B}} B = \frac{T_{\textrm{m}}}{T_{\textrm{e}} - T_{\textrm{m}}}\Delta \mu _{\textrm{s}}^{\textrm{b}}, \end{aligned}$$

where $\mu _{\textrm{s}}^{\textrm{b}}$ corresponds to the spin voltage blocking the magnon current. Introducing the Carnot efficiency of the system, $\eta _C = 1 - T_{\textrm{e}}/T_{\textrm{m}}$, Eq. (38) takes the form,

39 $$\begin{aligned} -\frac{\Delta \mu _{\textrm{s}}^{\textrm{b}}}{g\mu _{\textrm{B}} B} = \eta _C. \end{aligned}$$

Equation (38) can also be rewritten using the spin Seebeck coefficient taken at $T=T_{\textrm{m}}$, $S_s = \frac{g \mu _{\textrm{B}} B}{T}|_{T=T_{\textrm{m}}}$^68^,

40 $$\begin{aligned} \Delta \mu _{\textrm{s}}^{\textrm{b}} = -\Delta T S_s \big |_{\scriptscriptstyle T = T_{\textrm{m}}}. \end{aligned}$$

From Eq. (40), one can infer that the thermally generated spin current flows until the applied spin voltage to the FM electrode compensates the spin voltage associated with the spin Seebeck effect. Notice that, Eq. (40) leads to the correct linear response regime result, i.e. in the limit $\Delta T\rightarrow 0$, $T_{\textrm{m}}\rightarrow T_0$ and $S_S\rightarrow \frac{g \mu _{\textrm{B}} B}{T_0}$ ^68^.

Furthermore, for vanishing spin/magnon current, the efficiency reads

41 $$\begin{aligned} \eta = -\frac{\Delta \mu _{\textrm{s}}^{\textrm{b}}}{g\mu _{\textrm{B}} B} \end{aligned}$$

which is the Carnot efficiency given by Eq. (39). However, this situation is impractical, as there is no output power.

**Fig. 6:**
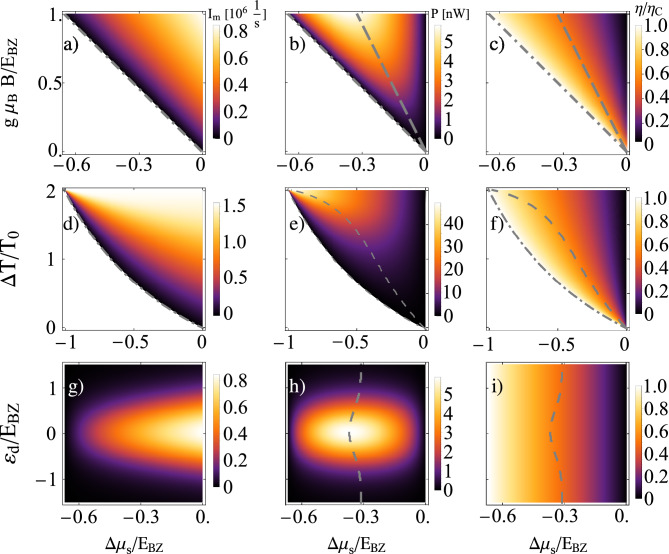
Spin current (**a,d,g**), power output (**b,e,h**) and efficiency (**c,f,i**) as a function of: the energy of transmitted magnons (first row), temperature bias (second row), dot’s level position (third row), the mean temperature (fourth row) and spin bias voltage. The dashed gray line in (**b,c**) indicates the maximum power [maximum efficiency], whereas the dash-dotted gray line indicates the blocking voltage. Only above this line, the system works as a heat engine. The other parameters are: (first row) $T_0 = 200$ K, $\Delta T = T_0$, $\varepsilon _{\textrm{d}} = 0$, (second row) $T_0 = 200$ K, $g\mu _{\textrm{B}}B = E_{\textrm{BZ}}$, $\varepsilon _{\textrm{d}} = 0$, (third row) $T_0 = 200$ K, $\Delta T = T_0$, $g\mu _{\textrm{B}}B = E_{\textrm{BZ}}$, (fourth row) $\Delta T = 50$ K, $g\mu _{\textrm{B}}B = E_{\textrm{BZ}}$, $\varepsilon _{\textrm{d}} = 0$ and $\Gamma _e = 0.01$ meV. The other parameters are the same as in Fig. 2.

Figure 6 presents the magnon current [a), d), g)], power [b), e), h)], and corresponding efficiency [c), f), i)] of the system as a function of the spin bias voltage on the horizontal axis and magnetic field, temperature bias, dot’s level position, and mean temperature on the vertical axis.

In each case, the power is a non-monotonic function of the spin bias voltage, $\Delta \mu _{\textrm{s}}$: it increases with decreasing $\Delta \mu _{\textrm{s}}$ until reaching a maximum, after which it decreases as the spin bias voltage continues to drop and finally vanishes for $\Delta \mu _{\textrm{s}}=\Delta \mu _{\textrm{s}}^{\textrm{b}}$. Considering power as a function of the transported magnon’s energy, $g\mu _\textrm{B}B$, and spin bias, shown in Fig. 6 b), the maximum value of *P* (for a given *B*) occurs at half the blocking spin bias voltage. Due to the linear increase of $\eta /\eta _C$ with $-\Delta \mu _s$, the efficiency reaches half of the Carnot efficiency when *P* is maximized. However, this holds only at sufficiently high temperatures, while for lower *T*, $\eta (P_{max})$ deviates from this value, as detailed in the following discussion of the maxima of the operational coefficients. Moreover, non-zero power can only be extracted for $\Delta \mu _{\textrm{s}}\in (\Delta \mu _{\textrm{s}}^{\textrm{b}},0)$. As the power is proportional to the magnon current, it follows similar behavior: it increases monotonically with $g\mu _{\textrm{B}}B$. The corresponding efficiency, shown in Fig. 6 c), increases linearly with decreasing spin bias voltage, achieving Carnot efficiency for $\Delta \mu _{\textrm{s}}^{\textrm{b}}$. However, at $\Delta \mu _{\textrm{s}}=\Delta \mu _{\textrm{s}}^{\textrm{b}}$ the power is zero as a result of vanishing magnon current.

Figure 6 e) depicts *P* as a function of the spin bias and the temperature difference $\Delta T$. In this case, the blocking spin voltage compensating the induced magnon current by the temperature difference $\Delta T/T_{0}$ satisfy the equation

42 $$\begin{aligned} \frac{\Delta T}{T_0} = \frac{-2\Delta \mu _{\textrm{s}}^{\textrm{b}}}{\Delta \mu _{\textrm{s}}^{\textrm{b}}+2g\mu _{\textrm{B}}B} \end{aligned}$$

derived using Eq. (38).

For a given value of the spin voltage $\Delta \mu _{\textrm{s}}$, the generated power *P* is an increasing function of the temperature difference $\Delta T$, which is a direct consequence of the increase in the magnon current with $\Delta T$. The corresponding efficiency, shown in Fig.  6 f), increases with decreasing spin bias voltage up to the Carnot efficiency, achieved at blocking spin voltage $\Delta \mu _{\textrm{s}}^{\textrm{b}}$. The efficiency at maximum power also increases monotonically with $-\Delta \mu _{\textrm{s}}$ reaching the Carnot efficiency for maximal temperature bias $\Delta T/T_{0} = 2$.

Figure 6 g) presents the magnon current $I_{\textrm{m}}$ as a function of the dot’s level position and the spin bias voltage. The magnon current is symmetrical with respect to $\varepsilon _{\textrm{d}}$ and is maximized when the dot’s energy level is situated at the Fermi level of metallic electrode. Beyond specific values of $|\varepsilon _{\textrm{d}}|$, the magnon current becomes strongly suppressed, as explained in the previous section. The corresponding output power, shown in Fig. 6 h), is a non-monotonic function of both $\varepsilon _{\textrm{d}}$ and $\mu _{\textrm{s}}$. The maximum of *P* for a given spin bias voltage always occurs at $\varepsilon _{\textrm{d}}=0$, which directly results from the aforementioned maximization of the magnon current. In turn, the efficiency, presented in Fig. 6 i), does not depend on $\varepsilon _{\textrm{d}}$. As a consequence, the devices operates at maximum power (for a given $\varepsilon _{\textrm{d}}$) with approximately half of the Carnot efficiency, $\eta \approx 0.5 \eta _{\textrm{C}}$, regardless of the dot’s energy level. Moreover, the exact value of $\eta$ increases with increasing maximum power output, which depends on $\varepsilon _{\textrm{d}}$.

**Fig. 7:**
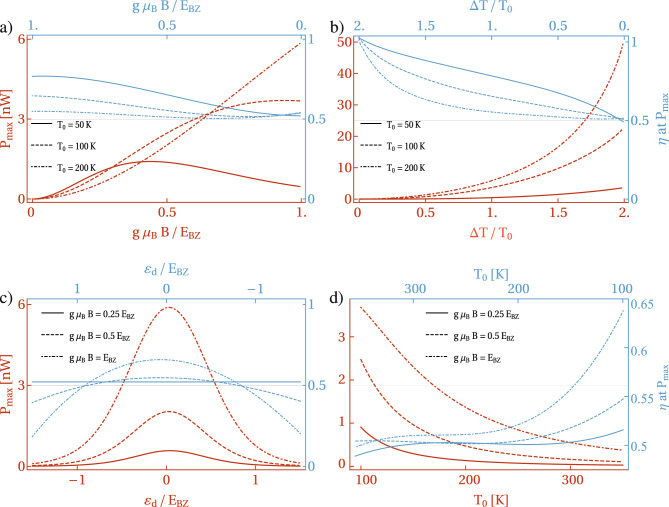
Maximum power (red lines) and efficiency at maximum power (blue lines) as a function of the energy of the transported magnons (**a**), the temperature bias (**b**) for indicated values of the mean temperature, the dot’s level position (**c**) and the mean temperature (**d**) for indicated values of the energy of transported magnons and $\Delta T = 50$ K. The efficiency is scaled in Carnot efficiency $\eta _{\textrm{c}}$. The other parameters are the same as in Fig. 6.

Notice that, from a practical perspective, it is not the power at maximum efficiency but rather the maximum power and its corresponding efficiency, that are important. Therefore, we focus on these quantities in the following discussion. In Fig. 7 a) we show the maximum power $P_{\textrm{max}}$ and its corresponding efficiency as a function of magnon energy (magnetic field). For temperatures $T_0\le 100$ K, the maximum power, $P_{\textrm{max}}$, increases with magnon energy until it reaches a maximum at a specific value of the magnetic field $g\mu _B B$, and then decreases with $g\mu _B B$. Simultaneously, its maximum shifts towards larger values of $g\mu _B B$ with increasing $T_0$, and for sufficiently large temperatures, $P_{\textrm{max}}$ monotonically rises with $g\mu _B B$, reaching a maximum at the maximal available value of acoustic magnon energy. In contrast, the corresponding efficiency, $\eta (P_{\textrm{max}})$, at high temperatures almost does not depend on magnon energy, taking the value equal to 0.5. Generally, $\eta (P_{\textrm{max}})$ achieves nearly 0.5 regardless of $T_0$, and rises with the magnetic field, more prominently at lower temperatures. One can notice that at lower temperatures, better optimization between maximum power and its efficiency is achieved. However, at higher temperatures, much larger maximum power can be extracted with satisfactory efficiency, $\eta (P_{\textrm{max}})$.

Figure 7 b) illustrates $P_{\textrm{max}}$ and $\eta (P_{\textrm{max}})$ responses to an applied temperature difference $\Delta T$. Both quantities increase with $\Delta T$, with the efficiency approaching the Carnot efficiency at the maximal temperature difference $\Delta T/T_0 = 2$. We point out that $\eta (P_{\textrm{max}})$ reaches Carnot efficiency at $\Delta T/T_0 = 2$ regardless of the mean temperature $T_0$. Figure 7c) presents these quantities as a function of the dot’s energy level $\varepsilon _{\textrm{d}}$, revealing that $P_{\textrm{max}}$ achieves its maximum for the dot’s energy level situated near the Fermi energy of the metallic lead. The peak of $P_{\textrm{max}}$ is slightly shifted from $\varepsilon _{\textrm{d}} = 0$ and moves toward positive values of $\varepsilon _{\textrm{d}}$ with decreasing magnetic field. The corresponding efficiency is close to 0.5 in the presented range of $\varepsilon _{\textrm{d}}$ for smaller magnetic fields. However, for larger $g\mu _B B$, the efficiency $\eta (P_{\textrm{max}})$ exceeds 0.5 over a broad range of the dot’s energy level around $\varepsilon _{\textrm{d}} = 0$, where the maximum power is also most remarkable.

For completeness, Fig. 7d) shows that both $P_{\textrm{max}}$ and its efficiency, calculated for a fixed temperature bias, decrease as the mean temperature $T_0$ increases. Therefore, the best optimization is achieved when $\Delta T > T_0$, as confirmed by the results shown in Fig. 7b). In conclusion, the results presented throughout this section clearly indicate the robustness of the proposed QD-based hybrid spin Seebeck engine.

## Conclusion

In conclusion, we provided a thorough analysis of spin caloritronics in a QD-based hybrid with a MI, focusing on exploring the temperature-induced effects and designing the system to operate as a spin Seebeck heat engine with optimal performance.

We also investigated the influence of the energy-dependent density of states and magnon-magnon interactions on thermally generated spin transport. We demonstrated that many-body magnon interactions significantly enhance magnon transport by increasing their density of states. Additionally, we examined the effects of various factors, including the Landé factor of the QD, the spin-polarization of the metal, and the system’s temperature.

Our results show that for the Landé factor of the QD being smaller than that of the MI, magnon current cannot flow. Conversely, for $g \gg g_{mag}$, the influence of the magnetic field on the magnon dispersion becomes negligible, and the magnon current is maximized. Furthermore, the considered system performs best as a spin current generator when the metallic electrode is nonmagnetic, as any non-zero spin polarization of the lead negatively impacts the magnitude of the spin current. We also found a strong spin current rectification effect due to an asymmetric response of the system to the applied temperature bias. Moreover, we showed that the system operates as a spin diode when the energy level of the QD is offset from the Fermi energy of the metallic electrode, with the rectification factor increasing as the QD energy level moves further from the Fermi energy.

Finally, we proposed the considered system as a device operating as a spin Seebeck engine. We established that high power can be extracted with relatively high efficiency, exceeding half of the Carnot efficiency.

Our findings contribute to the growing understanding of spin caloritronics as a promising avenue for future computational and renewable energy generation technologies. Additionally, the obtained results can pave the way for designing spin caloritronics experiments and help interpret their outcomes.

## Supplementary Information

Supplementary Information.

## Data Availability

The datasets used and/or analysed during the current study available from the corresponding author on reasonable request.
