# Supplementary material for: Spin caloritronics of a quantum dot coupled to a magnetic insulator and normal metal
Source: Sci Rep. 2025 Jul 2;15:23208. doi: 10.1038/s41598-025-04413-6 (PMC12223087; doi:10.1038/s41598-025-04413-6)
Supplement: Supplementary file 1 — Supplementary Information. [file 41598_2025_4413_MOESM1_ESM.pdf]

Spin caloritronics of a quantum dot coupled to a magnetic insulator and normal metal

Emil Siuda[1,*] **and Piotr Trocha**[1]

[1]Institute of Spintronics and Quantum Information, Faculty of Physics and Astronomy, Adam Mickiewicz University, Poznań, 61-614, Poland
[*]emisiu@amu.edu.pl

Density of states and magnon current at various temperatures

Figure S1 shows the DOS calculated using the SP and LWL models for various temperatures. As the temperature increases, the renormalization factor decreases, which, in turn, enhances the DOS. As the system approaches room temperature, magnon-magnon interactions become increasingly relevant, especially for high-energy magnons. This behavior is also evident in the magnon current shown in Fig.S3. In the low-temperature regime, as shown in Figs.S3a) and b), magnon-magnon interactions do not significantly affect the magnon current. However, at higher temperatures, they play a more significant role, enhancing the magnon current. In both cases, the constant density-of-states model (red) significantly overestimates the magnon current. However, this discrepancy diminishes at higher temperatures.

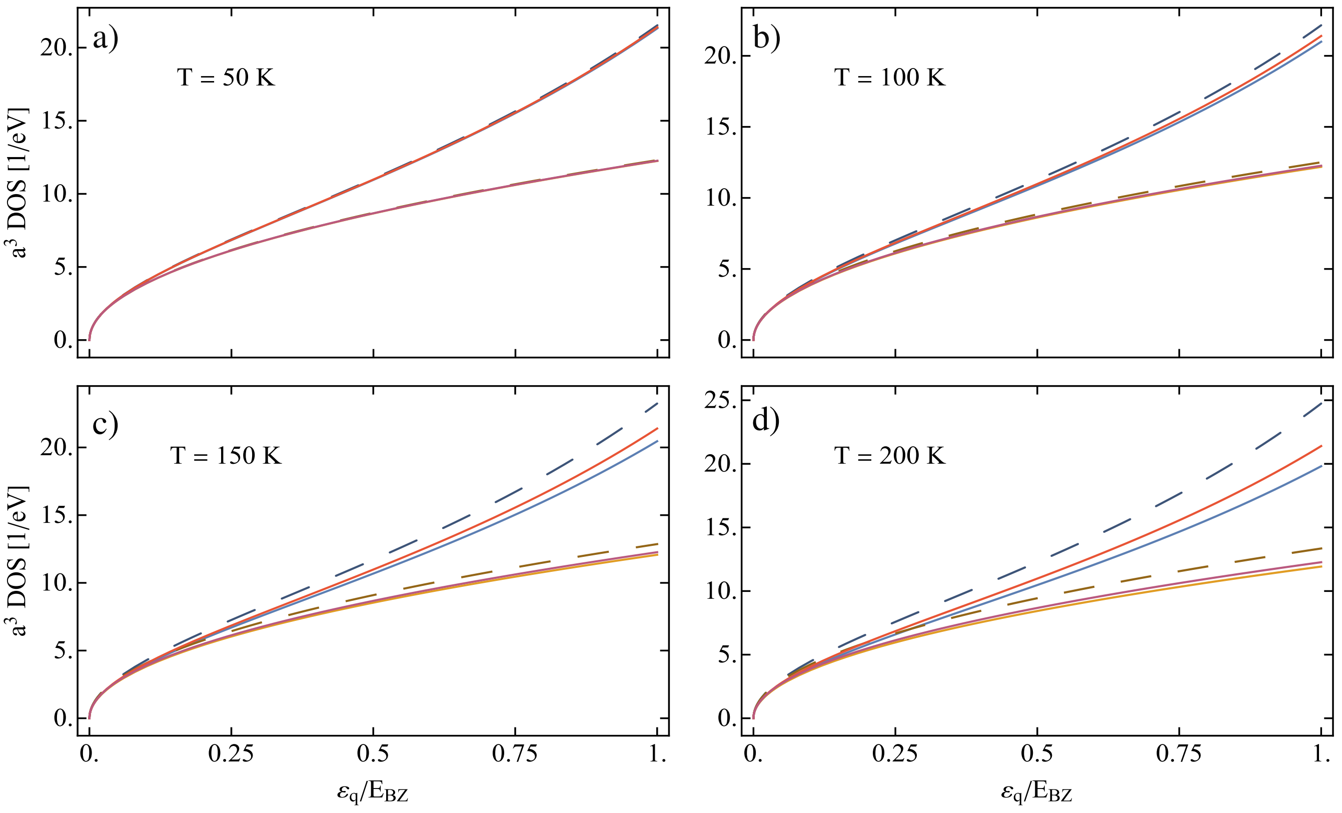

Figure S1. The DOS of a magnetic insulator as a function of magnon energy, calculated for the indicated temperature. Solid lines represent the case without magnon-magnon interactions ($\alpha = 1$), while dashed lines correspond to α calculated self-consistently, including magnon interactions.

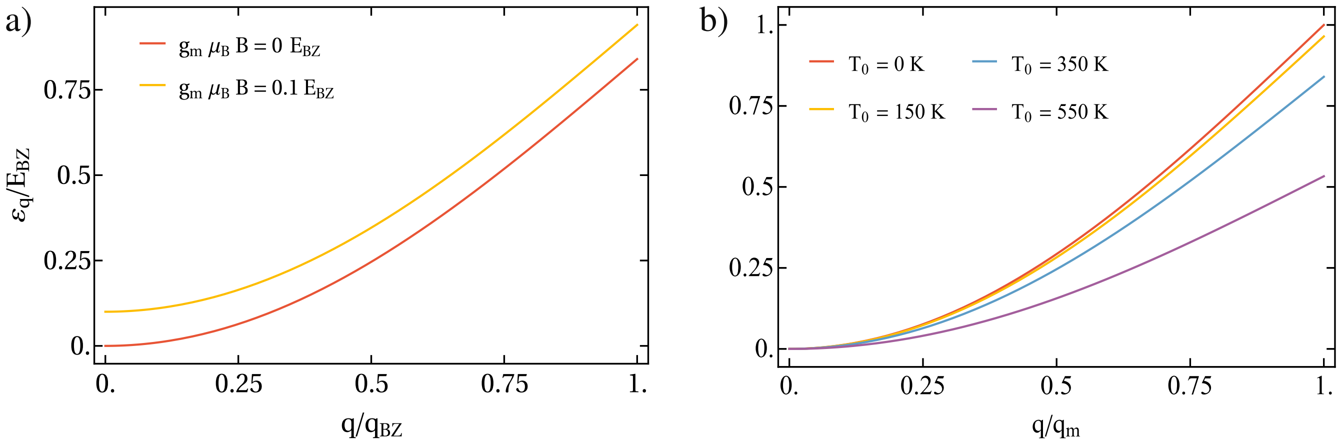

Figure S2. Comparison of dispersion relations calculated for a) the indicated values of the magnetic field applied to the MI, b) calculated for the indicated values of the temperature of the MI. In the panel a), the case of $g_m \mu_B B = 0 E_{\mathrm{BZ}}$ is equivalent to omitting the influence of the magnetic field on the magnons in the MI. The other parameters are the same as in the Fig. 2 in the main text.

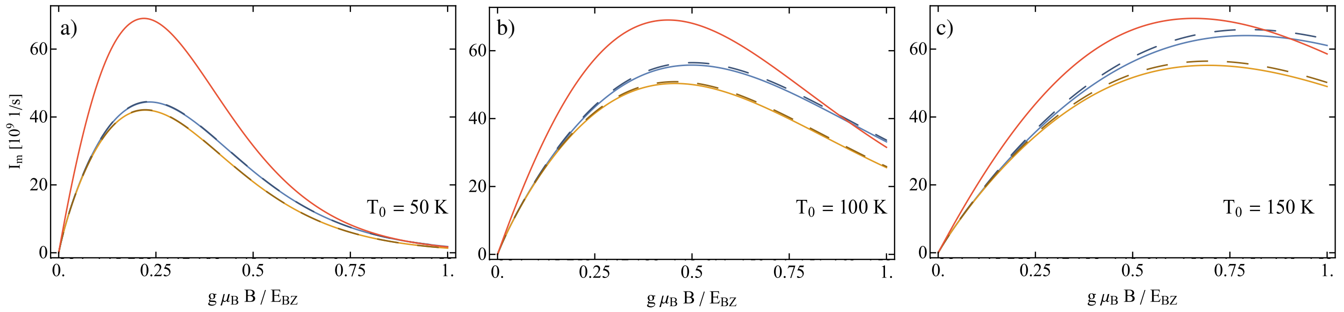

Figure S3. Magnon current as a function of $g\mu_{\mathrm{B}}B$ calculated for the indicated values of the temperature T_0. Solid lines represent the case without magnon-magnon interactions ($\alpha = 1$), while dashed lines correspond to α calculated self-consistently, including magnon interactions. For comparison, the red line shows the magnon current calculated using a constant density of states, $\rho_{\mathrm{m}} = const$.

Robustness of the spin diode effect

Figure S4 shows the rectification of the magnon current for various values of temperature and magnetic field. The spin diode effect, discussed in Sec. Rectification of the Current and Spin Diode Effect, remains robust under varying system parameters, occurring at both low and high temperatures, as well as under weak and strong magnetic fields. Lowering the mean temperature T_0 results in suppression of the magnon current when ε_{d} does not align with the Fermi level of the metal, especially for $\Delta T > 0$, as described in the section Rectification of the current and spin diode effect. This, in turn, yields a significantly rectified magnon current even for small values of ε_{d}. Similarly, decreasing the magnetic field narrows the range of ε_{d} for which the magnon current can flow when $\Delta T > 0$, resulting in rectification of the current. Even in the least favorable scenario, when both T_0 and B are high, rectification is still present for reasonable values of ε_{d}.

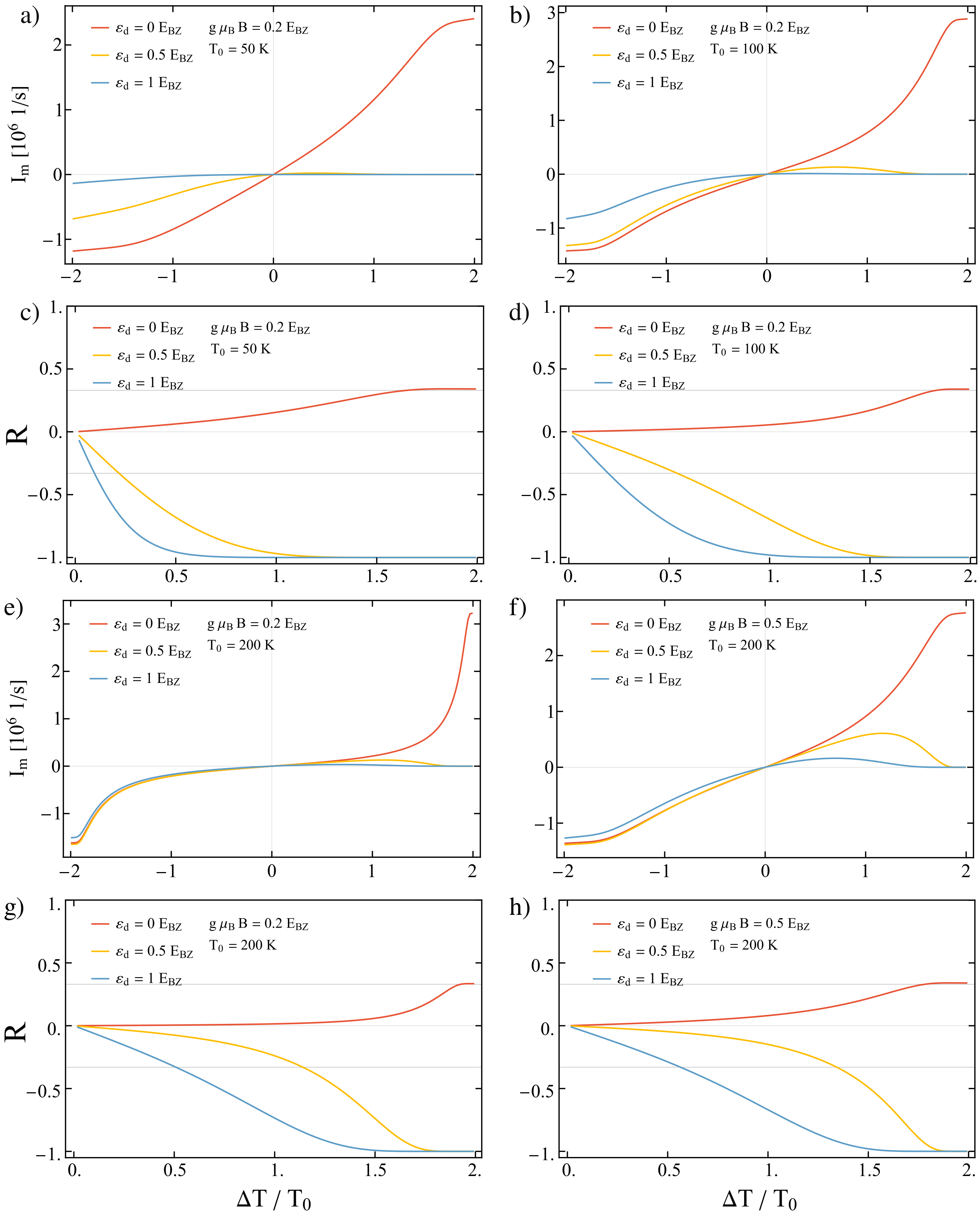

Figure S4. Magnon current and rectification coefficient as a function of the temperature difference between the reservoirs calculated for indicated values of the magnetic field B, mean temperature T_0, and dot's energy level ε_d. Other parameters as in Fig. 5 in the main text.
